# Supplementary material for: Quantitative Proteomic Study Unmasks Fibrinogen Pathway in Polycystic Liver Disease
Source: Biomedicines. 2022 Jan 27;10(2):290. doi: 10.3390/biomedicines10020290 (PMC8869147; doi:10.3390/biomedicines10020290)
Supplement: Supplementary file 1 [file biomedicines-10-00290-s001.zip › biomedicines-1534433-supplementary.pdf]

## Supplementary Material

*SupplementaryFigures*

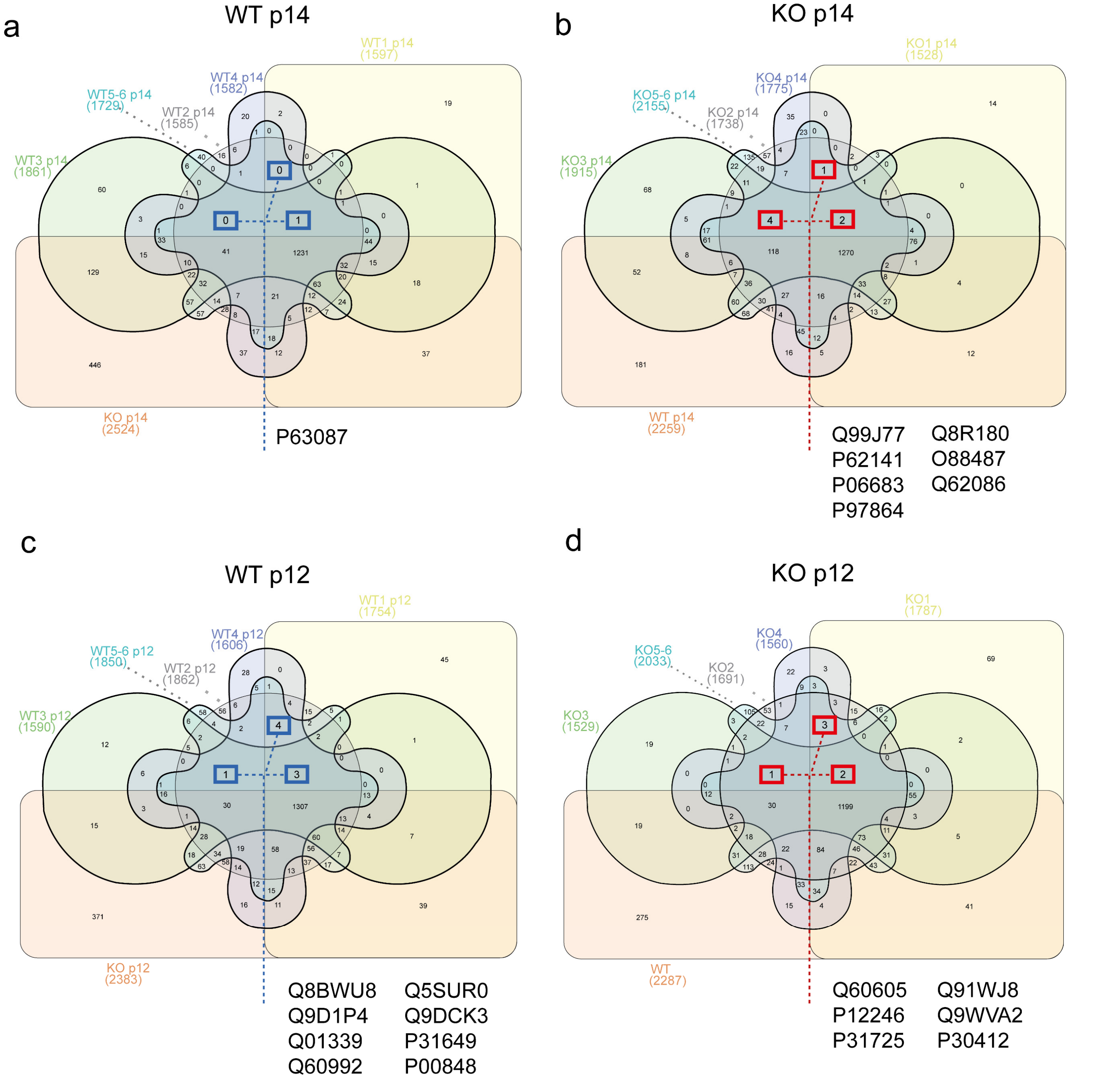


**Figure S1**. Qualitative DDA mass spectrometry analysis in the different groups of the study. Venn diagrams showing proteins present in 5/6 out of 6 independent samples in different and specific groups. List of proteins present in 5/6 out of 6 independent samples in *Wild Type* (WT) p14 group (**a**), in Mutant (KO) p14 group (**b**), in *Wild Type* (WT) p12 group (**c**), in Mutant (KO) p12 group (**d**).

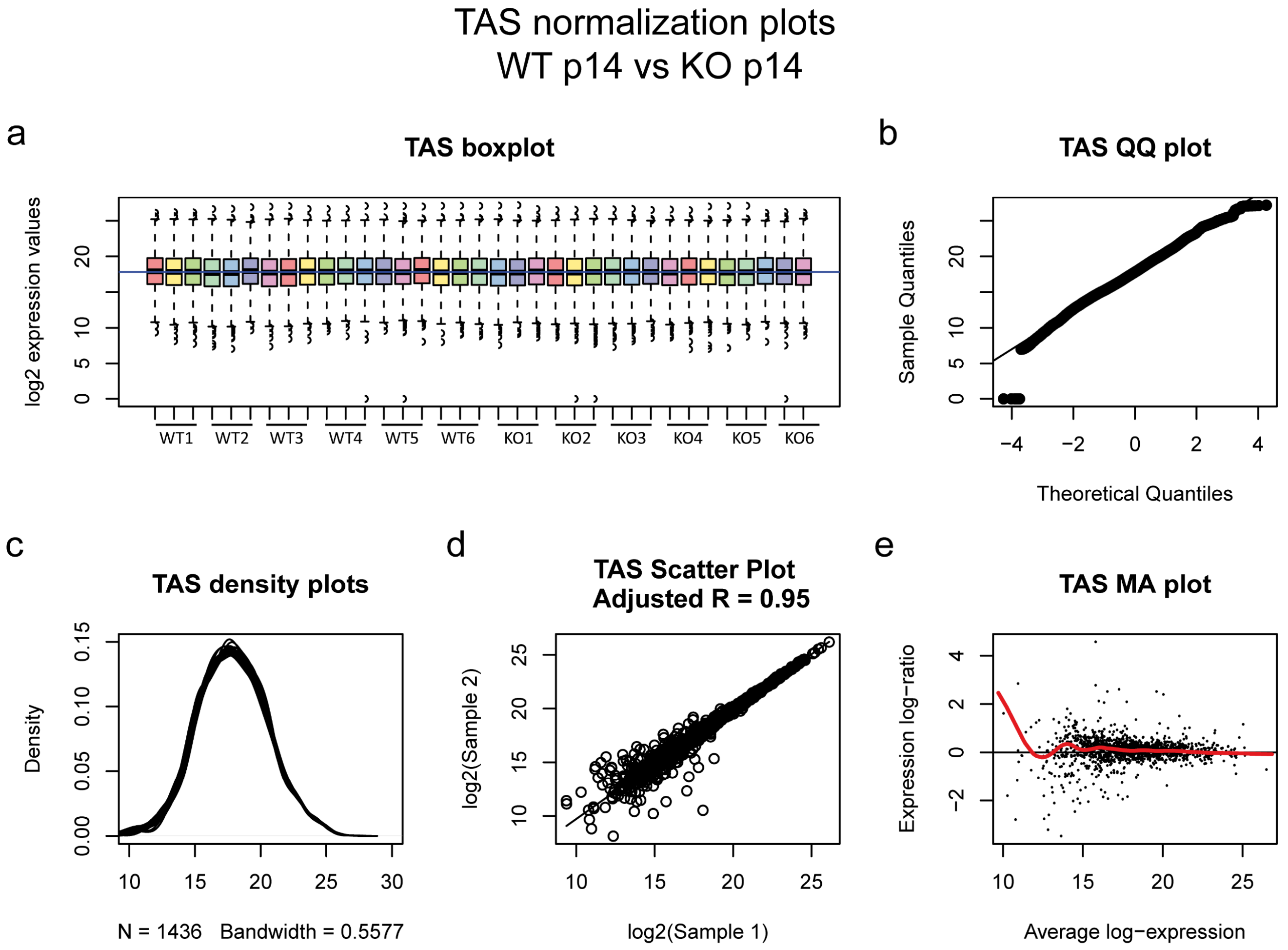


**Figure S2**. Total Area Sums (TAS) normalization plots p14 group. (**a**) TAS (Total Area Sums) boxplots show a graphical representation of log2 protein expression values (after TAS normalization). All samples, with its triplicates, are aligned with reference line. (**b**) TAS QQ plot comparing independent normal data on the vertical axis to a standard normal population on the horizontal axis. The linearity of points suggests that the data follows normal distribution. (**c**) TAS density plots are represented. The approximate representation of a Gaussian "bell" suggests that the data are normally distributed. Next, in order to determine the dispersion or distribution of our data we have performed two different analysis. (**d**) TAS scatter plot and correlation coefficient. Points dispersion and adjusted R with a value ≥0.95 (near to 1) suggest low dispersion. (**e**) TAS MA plot is shown. The slope of the line around 1 suggest good distribution of the analyzed data. In summary, these results show that data of p14 study follow a normal distribution and with a good distribution.

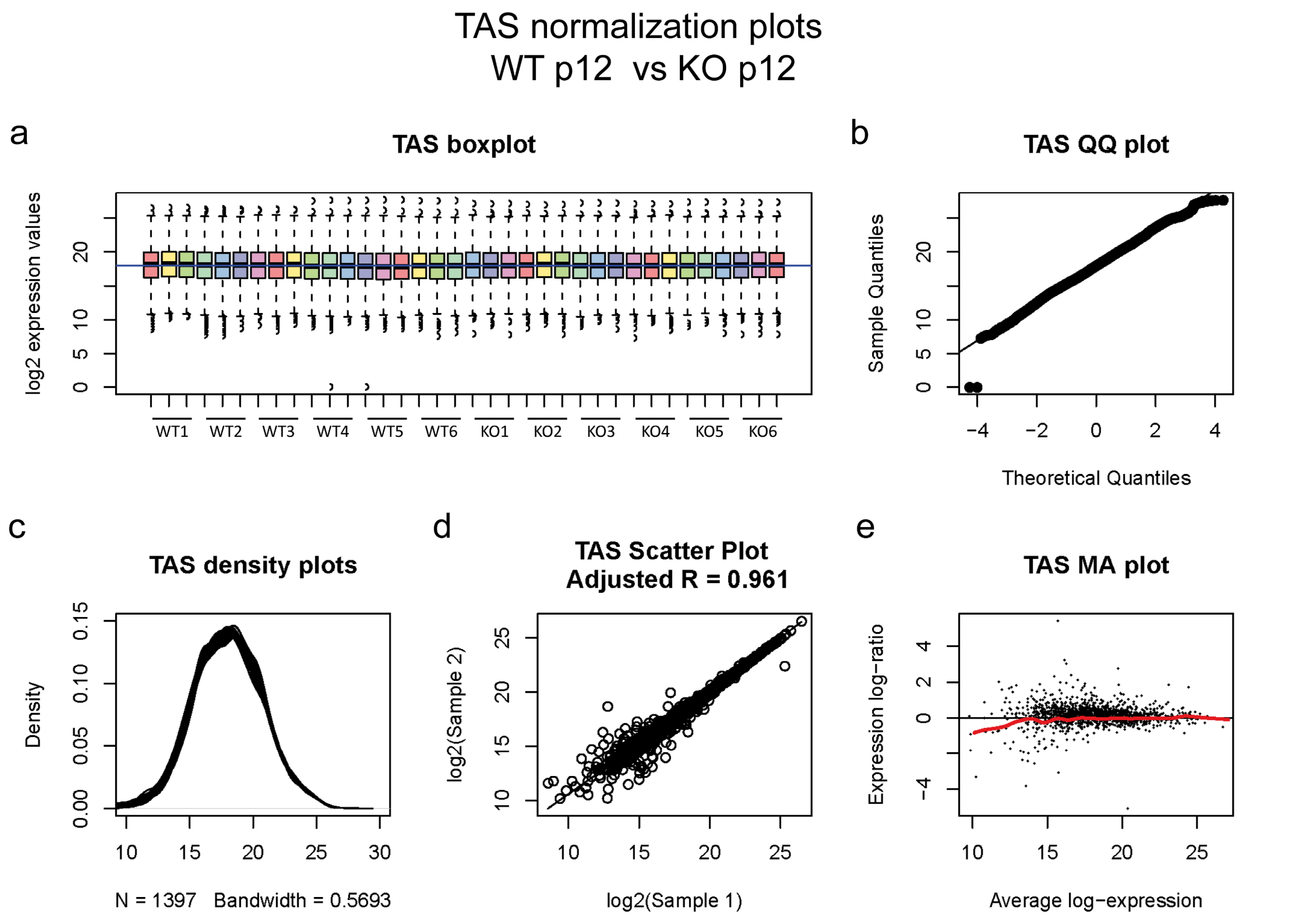


**Figure S3**. Total Area Sums (TAS) normalization plots of p12 group. (**a**) TAS boxplots show a graphical representation of log2 protein expression values (after TAS normalization). All samples, with its triplicates, are aligned with reference line. (**b**) The linearity of points in the TAS QQ plot suggests that the data follows normal distribution. (**c**) TAS density plots are represented. The approximate representation of a Gaussian "bell" suggests that the data are normally distributed. (**d**) TAS scatter plot and correlation coefficient. Points dispersion and adjusted R with a value ≥0.95 (near to 1) suggest a good distribution. (**e**) TAS MA plot is represented. The slope of the line around 1 suggest normalization of data. In conclusion, these results show that data of p12 study follow a normal distribution and with a good data dispersion.

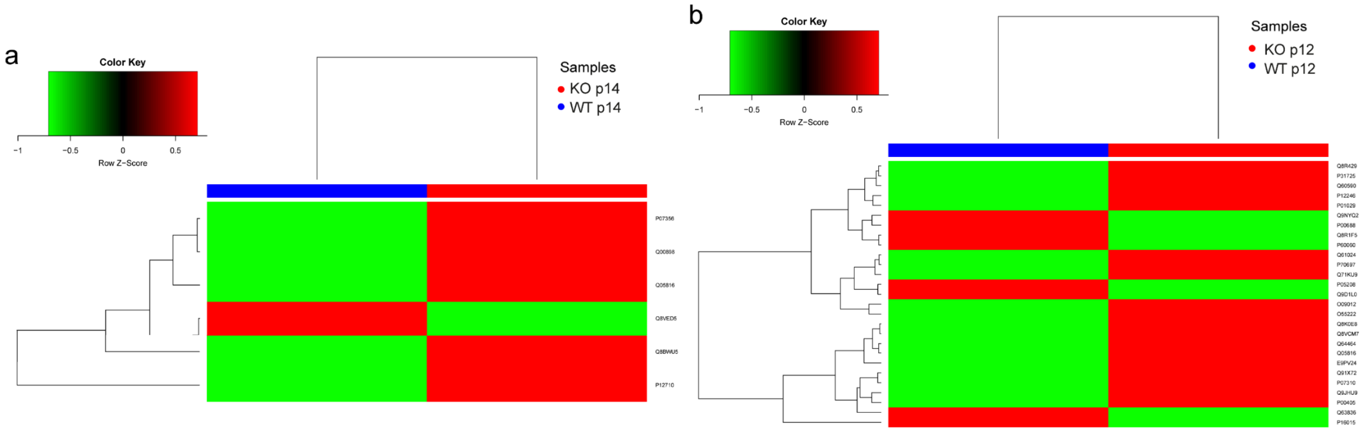


**Figure S4**. Heatmap figure of unsupervised cluster analysis of proteins with significant differences according to SWATH MS analysis. (**a-b**) Cluster of results corresponding to postnatal day 14 (a) and postnatal day 12 (b). Samples from the *Wild Types* (WT p12 and WT p14) and mutant (KO p12 and KO p14) groups are shown in blue and red, respectively. Color gradient is determined by the RowZ-score, where red and green represent increase and decrease, respectively, of protein abundance.

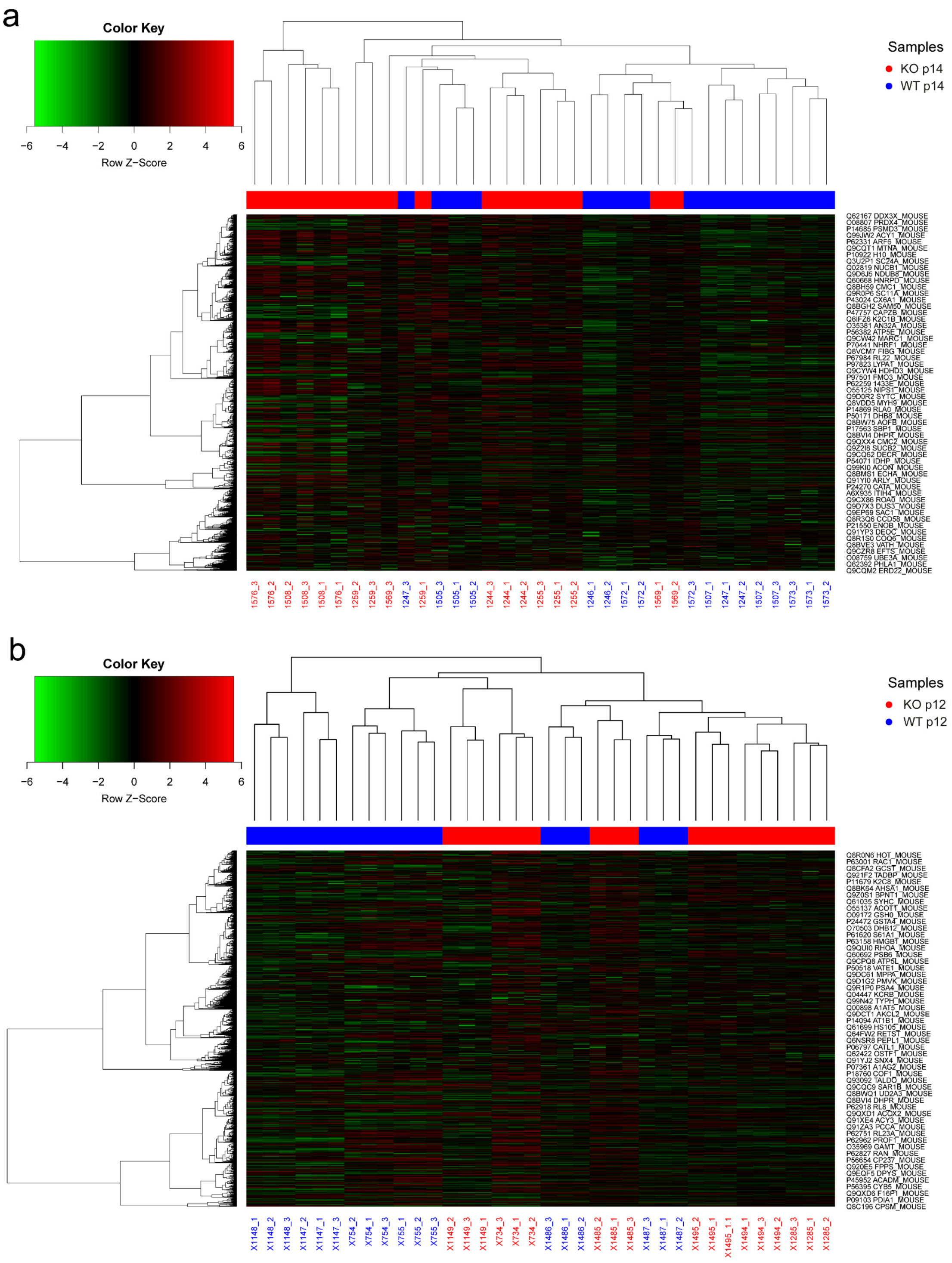


**Figure S5**. Unsupervised heatmap Cluster analysis of all proteins detected by SWATH-MS. Heatmap figure of cluster analysis of all proteins identified by SWATH MS. (**a-b**) Cluster of results corresponding to postnatal day 14 (p14) (a) and postnatal day 12 (p12) (b). Samples from the Wild Types (WT p12 and WT p14) and mutant (KO p12 and KO p14) groups are shown in blue and red, respectively. Color gradient is determined by the RowZ-score, where red and green represent increase and decrease, respectively, of protein abundance.

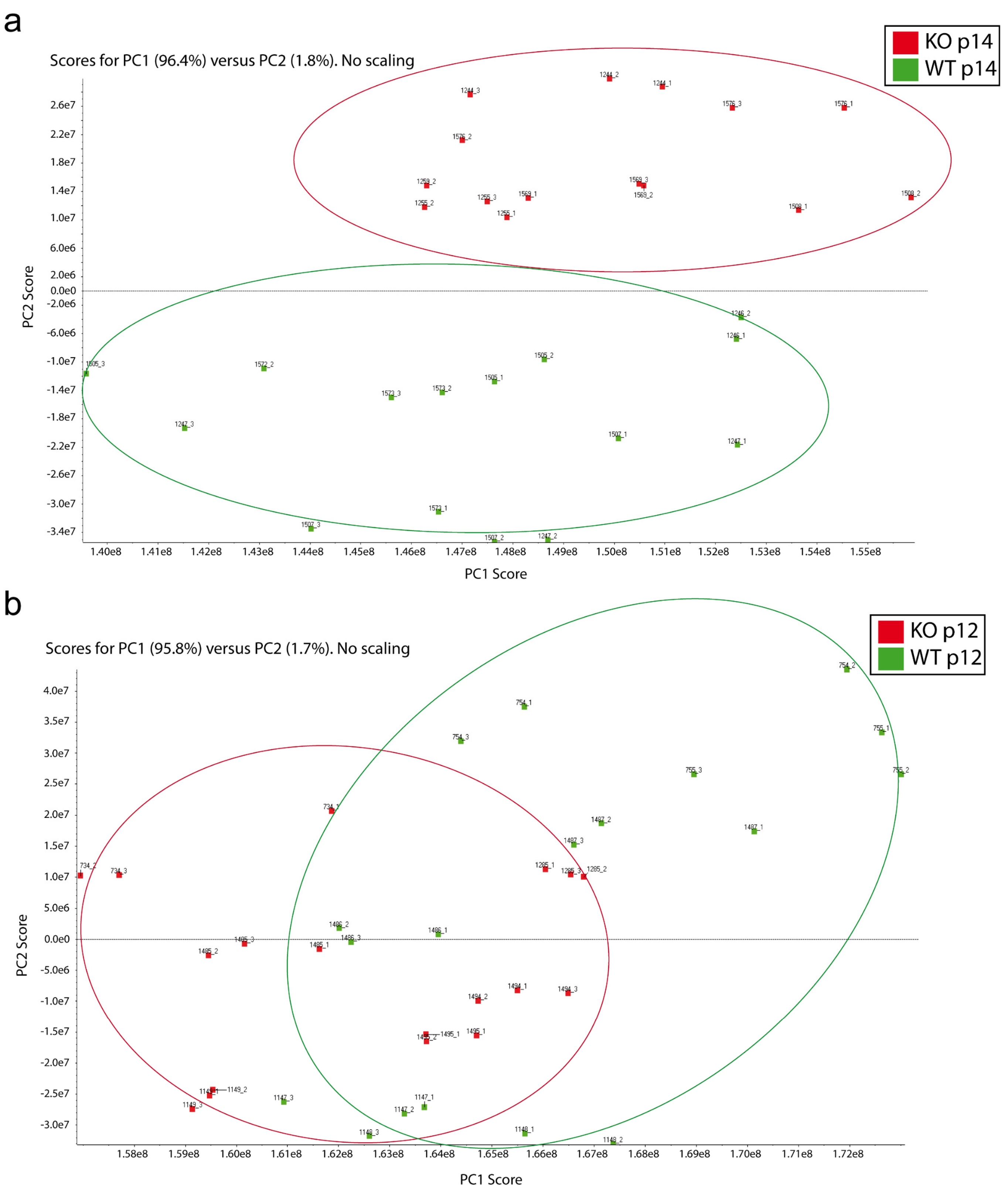


**Figure S6**. Unsupervised PCAs from SWATH MS data. (**a-b**) PCA multivariate statistical analysis of p14 (a) and p12 (b) data. Red and green circles represent KO (mutant) and WT (*Wild Types*) samples clusters, respectively. In p14 data, PCA has successfully separate the two clusters, however in p12 data we could observed overlap in part of samples (but we observed a separation tendency). In addition, PCA showed that the three technical replicates of each sample we clustered closely.

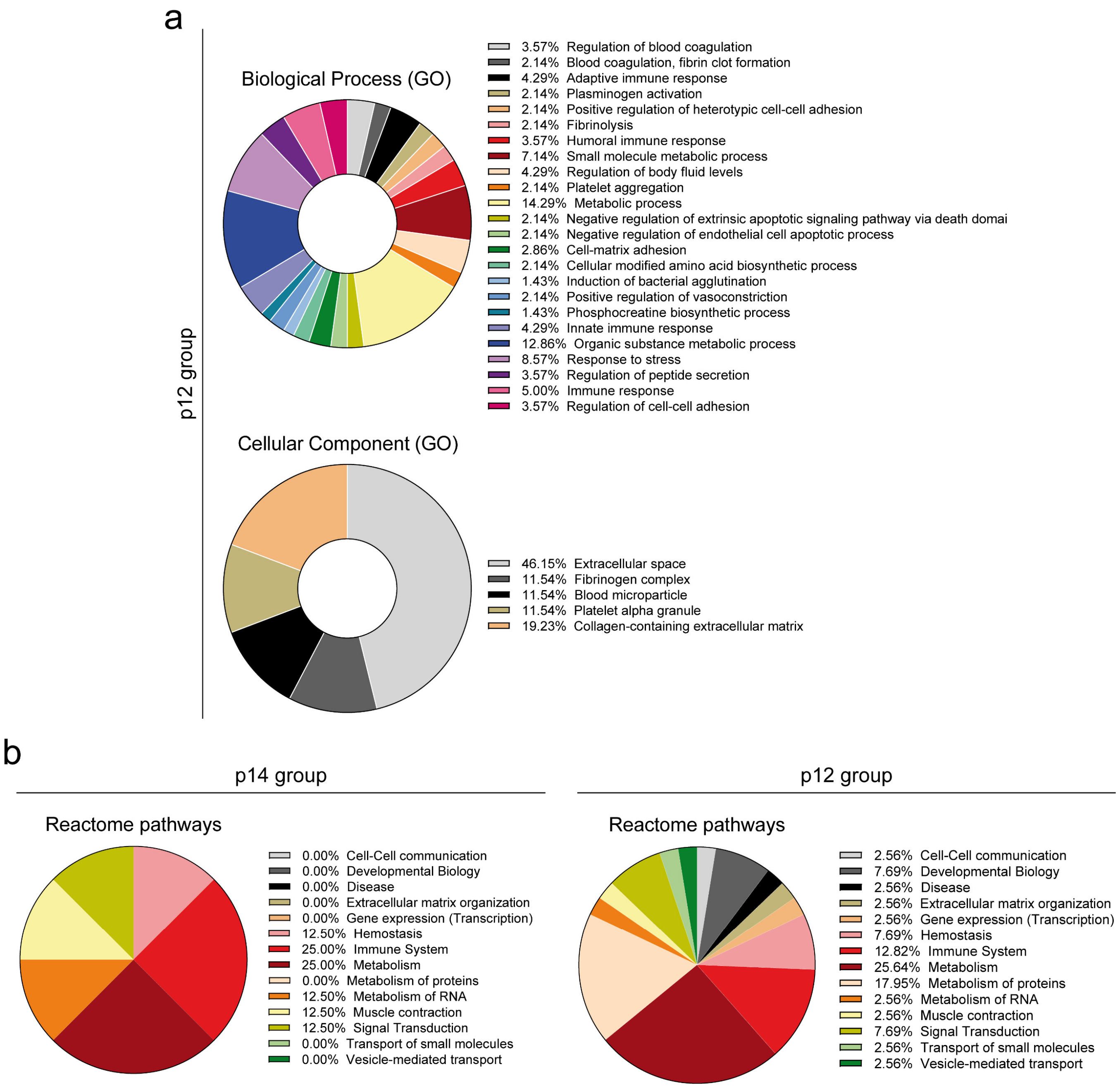


**Figure S7**. GO enrichment and pathway analysis of protein significantly regulated in hepatic cystogenesis according to String and Reactome, respectively. (**a**) show all Biological Process and Cellular components, with their respective percentages of proteins identified/quantify by SWATH approach in our analysis is respect to the database and according to the GO terms from String in p12 group. With respect to p14 group, the functional enrichment in the protein network did not detect significant enrichment differences. (**b**) Pathway analysis according to Reactome in p14 and p12 groups. All Reactome pathways are represented with their respective percentages with respect matched proteins. The pathways with cero matched proteins are represented as a value of 0.00%.

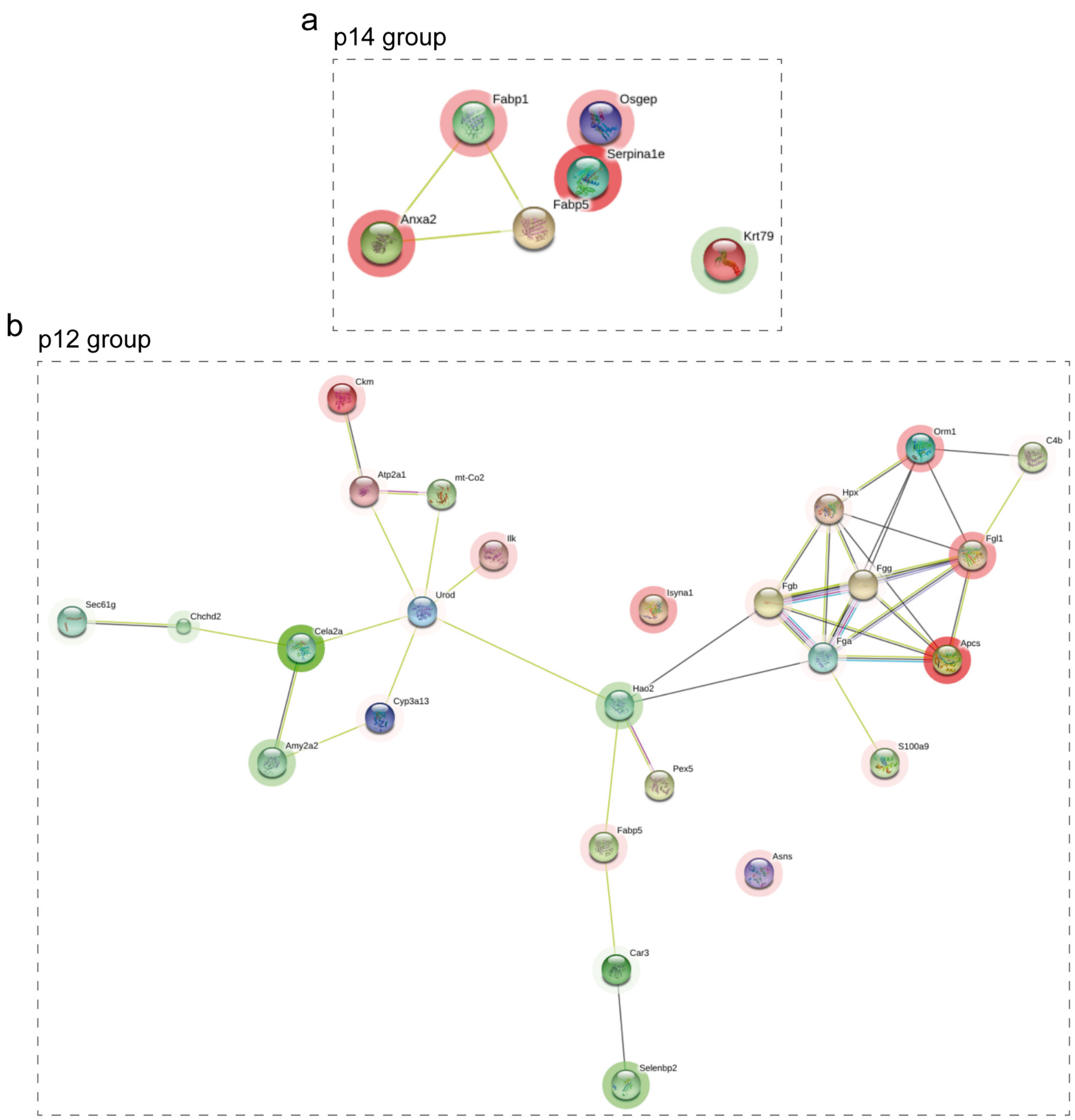


**Figure S8**. Full protein-protein interaction map according String. Proteins with significant increase and decrease protein abundance were submitted to String analysis. (**a**) and (**b**) Represents protein-protein interaction analysis of p14 and p12 data, respectively. Proteins are represented as nodules, which are colored red or green imply increase or decrease in protein abundance. Specifically, important selected clusters of proteins are represented in Figure 4.

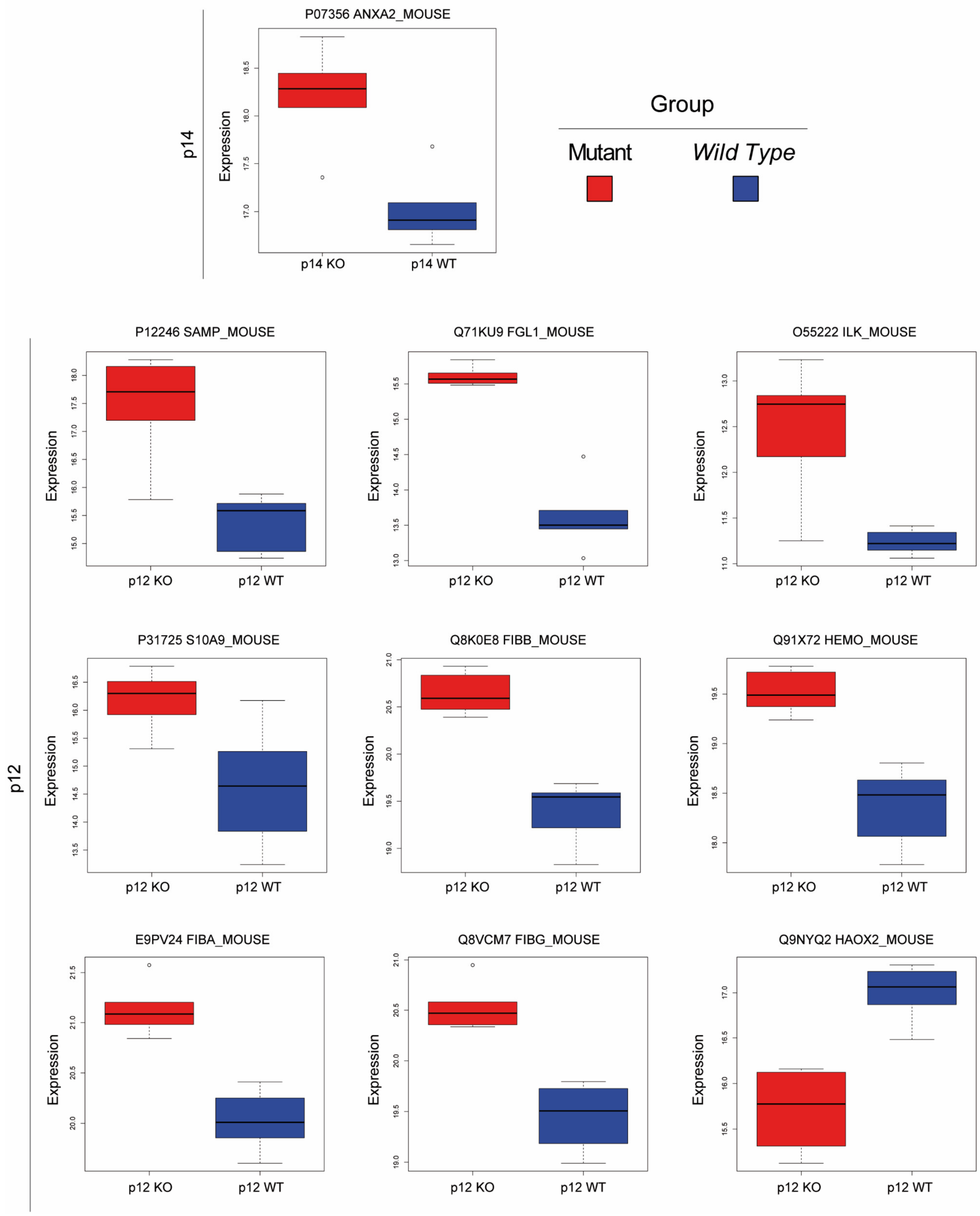


**Figure S9**. Box-plots of quantitative proteomic SWATH MS data of selected targets for validation. Box-plots represents protein abundance. The lower and upper ends of the box define the 25% and 75% quantiles, respectively, the middle line in the box is the median of the values and the whiskers are the 0% and 100% quantiles. Specific fold-change and adjusted p-value data was found in Table 1.

*Supplementary Tables*

**Table S1**. ID Analyzed samples, distribution in each group and samples pooling for the acquisition of shotgun runs to build the spectral library.

| Samples ID | Group | Secondary name | Pool for shotgun runs |
|---|---|---|---|
| 1246_1,2,3 | WT p14 | WT1 p14 | A |
| 1247_1,2,3 | WT p14 | WT2 p14 | A |
| 1505_1,2,3 | WT p14 | WT3 p14 | A |
| 1507_1,2,3 | WT p14 | WT4 p14 | A |
| 1572_1,2,3 | WT p14 | WT5 p14 | A |
| 1573_1,2,3 | WT p14 | WT6 p14 | A |
| 1244_1,2,3 | KO p14 | KO1 p14 | B |
| 1255_1,2,3 | KO p14 | KO2 p14 | B |
| 1259_1,2,3 | KO p14 | KO3 p14 | B |
| 1508_1,2,3 | KO p14 | KO4 p14 | B |
| 1569_1,2,3 | KO p14 | KO5 p14 | B |
| 1576_1,2,3 | KO p14 | KO6 p14 | B |
| 754_1,2,3 | WT p12 | WT1 p12 | C |
| 755_1,2,3 | WT p12 | WT2 p12 | C |
| 1147_1,2,3 | WT p12 | WT3 p12 | C |
| 1148_1,2,3 | WT p12 | WT2 p12 | C |
| 1486_1,2,3 | WT p12 | WT5 p12 | C |
| 1487_1,2,3 | WT p12 | WT6 p12 | C |
| 734_1,2,3 | KO p12 | KO1 p12 | D |
| 1149_1,2,3 | KO p12 | KO2 p12 | D |
| 1285_1,2,3 | KO p12 | KO3 p12 | D |
| 1485_1,2,3 | KO p12 | KO2 p12 | D |
| 1494_1,2,3 | KO p12 | KO5 p12 | D |
| 1495_1,2,3 | KO p12 | KO6 p12 | D |

**Table S2**. Primers sequence list used for RT-qPCR analysis. *Gapdh* (Glyceraldehyde 3-phosphate dehydrogenase); *Anxa2* (Annexin A2); Apcs (Amyloid P Component, Serum); *Fgl1* (Fibrinogen Like 1); *Ilk* (Integrin Linked Kinase); *S100a* (S100 Calcium Binding Protein A1); *Fgb* (Fibrinogen Beta Chain); *Hpx* (Hemopexin); *Fga* (Fibrinogen Alpha Chain); *Fgg* (Fibrinogen Gamma Chain); *Hao2* (Hydroxyacid Oxidase 2).

| Gene name | Primer sequence (5′-3′) |
|---|---|
| Housekeeping | |
| *Gapdh* | Forward 5′-ATTTGCCGTGAGTGGAGTCAT-3′<br>Reverse 5′-GCAAAGTGGAGATTGTTGCCA-3′ |
| p12 group | |
| *Anxa2* | Forward 5′-ACGAAATCCTGTGCAAGCTC-3′<br>Reverse 5′-TCTCAGCATCGAAGTTGGTG-3′ |
| p12 group | |
| *Apcs* | Forward 5′-TGTTTGTCTTCACCAGCCTTC-3′<br>Reverse 5′-GATGTGGGATCAGCTTCACA-3′ |
| *Fgl1* | Forward 5′-CCCTCGAGAGTGAGAACTGC-3′<br>Reverse 5′-GCTGTGCAATCATGGTCTGT-3′ |
| *Ilk* | Forward 5′-TGGAGCACGGATTAATGTGA-3′<br>Reverse 5′-TGCATTGATGTCAGCCTTGT-3′ |
| *S100a* | Forward 5′-AGGACCTGGACACAAACCAG-3′<br>Reverse 5′-GGGTTGTTCTCATGCAGCTT-3′ |
| *Fgb* | Forward 5′-AAGGCTACTGCCAACCAGAA-3′<br>Reverse 5′-CCCGTAGGACACAACACTCC-3′ |
| *Hpx* | Forward 5′-CCGAACACTGCTTGGATACC-3′<br>Reverse 5′-AGTGACCCCTCCACACAAAC-3′ |
| *Fga* | Forward 5′-GTACGTGGCCCAAGAGTTGT-3′<br>Reverse 5′-CTGAAGGGCATTTGTGGTTC-3′ |
| *Fgg* | Forward 5′-TTCGGTAGTTTCTGCCCAAC-3′<br>Reverse 5′-TTTCAGCCCGGAATAAGATG-3′ |
| *Hao2* | Forward 5′-CAATGACAACTTGGCAGCAT-3′<br>Reverse 5′-ATCTCCTGCCCTTGGATTGT-3′ |

**Table S3.** List of proteins presented in 5/6 out of 6 independent samples of different groups after qualitative DDA analysis. First, second and third columns represented the protein accession, protein code and gene name respectively. Fourth column represented the presence (in 6 or 5 samples) of the different proteins in WT (*Wild Types*) or KO (Mutant) groups. Fifth and sixth column represent the Fold change and p-value of quantitative SWATH analysis in the proteins founded by DDA analysis.

| Protein accession[1] | Protein code | Gene name | DDA (WT/KO) | SWATH Fold-change WT to KO | SWATH p-value (p≤0.05 student's test) |
|---|---|---|---|---|---|
| | | *Wild Type* p14 group | | | |
| P63087 | PP1G | *Ppp1cc* | 5/0 | - | - |
| | | Mutant p14 group | | | |
| Q99J77 | SIAS | *Nans* | 0/6 | 1,15 | 0,0065 |
| P62141 | PP1B | *Ppp1cb* | 0/5 | 1,12 | 0,0479 |
| P06683 | CO9 | *C9* | 0/5 | - | - |
| P97864 | CASP7 | *Casp7* | 0/5 | - | - |
| Q8R180 | ERO1A | *Ero1a* | 0/5 | 1,37 | 0,0457 |
| O88487 | DC1I2 | *Dync1i2* | 0/5 | - | - |
| Q62086 | PON2 | *Pon2* | 0/5 | 1,14 | 0,3607 |
| | | *Wild Type* p12 group | | | |
| Q8BWU8 | T2L1 | *Etnppl* | 6/0 | - | - |
| Q9D1P4 | CHRD1 | *Chordc1* | 6/0 | - | - |
| Q01339 | APOH | *Apoh* | 5/0 | - | - |
| Q60992 | VAV2 | *Vav2* | 5/0 | - | - |
| Q5SUR0 | PUR4 | *Pfas* | 5/0 | 1,45 | 0,2577 |
| Q9DCK3 | TSN4 | *Tspan4* | 5/0 | - | - |
| P31649 | S6A13 | *Slc6a13* | 5/0 | - | - |
| P00848 | ATP6 | *Mtatp6* | 5/0 | - | - |
| | | Mutant p12 group | | | |
| Q60605 | MYL6 | *Myl6* | 0/6 | 1,29 | 0,0003 |
| P12246 | SAMP | *Apcs* | 0/6 | 4,37 | 4,79E-07 |
| P31725 | S10A9 | *S100a9* | 0/5 | 2,39 | 1,45E-06 |
| Q91WJ8 | FUBP1 | *Fubp1* | 0/5 | - | - |
| Q9WVA2 | TIM8A | *Timm8a1* | 0/5 | - | - |
| P30412 | PPIC | *Ppic* | 0/5 | - | - |

[1] Protein accession according to Uniprot.
